# Change in timed walk as primary outcome measure of treatment response in HAMLET-P: HAM/TSP MuLticentre Efficacy trial-Prednisolone

**DOI:** 10.1186/1742-4690-11-S1-P30

**Published:** 2014-01-07

**Authors:** Fabiola Martin, Eisuke Inoue, Maria Fernanda Rios Grassi, Ramon de Almeida Kruschewsky, Irene Cortese, Marcus T Silva, Steve Jacobson, Bernardo Galvão-Castro, Yoshihisa Yamano, Graham Taylor, John Martin Bland

**Affiliations:** 1 Centre for Immunology and Infection, Department of Biology, Hull and York Medical School, University of York, York, UK; 2 School of Pharmacy, Kitasato University, Tokyo, Japan; 3 Advanced Laboratory of Public Health, Gonçalo Moniz Center, Fundação Oswaldo Cruz, Salvador, Bahia, Brazil; 4 Bahiana School of Medicine and Public Health (EBMSP) Salvador, Bahia, Brazil; 5 Viral Immunology Section, NINDS/NIH, Bethesda, MD, USA; 6 Clinical Research Laboratory on Neuroinfection Diseases Instituto de Pesquisa Clínica Evandro Chagas Fundação Oswaldo Cruz, Fiocruz, Brazil; 7 Department of Rare Diseases Research, Institute of Medical Science, St. Marianna University School of Medicine, Kanagawa, Japan; 8 Section of Infectious Diseases, Faculty of Medicine, Imperial College London, London, UK; 9 Department of Health Sciences, University of York, York, North Yorkshire, UK

## Background

In the absence of an internationally recognised biomarker of treatment response in HAM/TSP, the HAM/TSP clinial trial study group chose improvement of 10 meter time walk (TW) as the primary outcome measure for the HAMLET-P trial.

## Aim

To define the minimum change in TW required for an observed treatment effect to be detectable and important. To calculate the sample size required for 90% power to detect this difference.

## Methods

Prospectively collected TW (seconds/10m) of HAM/TSP patients from the four countries were submitted to two biostatisticians (Japan+UK). Analysis of covariance and log transformed TW were used.

## Results

Matched TW data (baseline+6 months) were available for a total of 76 patients. Mean (SD,median) TW were 23.46 (±18.9,16.32) at baseline, 24.85 (±23.89,16.38) at 6 months. Mean (SD,median) log10m TW were 2.89 (±0.72,2.79) at baseline, 2.91 (±0.74,2.80) at 6 months. The estimated SD of log10m TW after adjustment for the baseline measurement was 0.26. With 30 participants/group, we have 90% power to detect a difference of ±0.21. This corresponds to a ratio of 0.81 or 1.23, so we could detect a decrease in time of 19% or an increase of 23%. With power 80% we could detect a difference of -15% or +18%.

## Conclusions

Prospectively collected longitudinal data on TW is useful in measuring inter- and intra- patient variability of this clinical efficacy marker. To power HAMLET-P at 90%, a minimum of 30 patients are needed in each arm, to be increased by 3-5 patients/arm to cover for 10-15% estimated trial drop-out rate.

